# NMR Structural Study of Syndecan-4 Transmembrane Domain with Cytoplasmic Region

**DOI:** 10.3390/molecules28237855

**Published:** 2023-11-29

**Authors:** Minseon Kim, Yongae Kim

**Affiliations:** Department of Chemistry, Hankuk University of Foreign Studies, 81 Oedae-ro, Mohyeon, Yongin 17035, Republic of Korea; alstjs032@naver.com

**Keywords:** syndecan-4, Syd4-eTC, PIP_2_, solid-state NMR, transmembrane protein

## Abstract

Syndecan-4 (SDC4) consists of transmembrane heparan sulfate proteoglycan (HSPG) belonging to the syndecan family. It is present in most cell types of Mammalia. Its structure contains a heparan-sulfate-modified extracellular domain, a single transmembrane domain, and a short C-terminal cytoplasmic domain. Regarding the overall cellular function of SDC4, other cells or ligands can bind to its ecto-domain. In addition, 4,5-bisphosphate phosphatidylinositol (PIP_2_) or protein kinase Cα can bind to its cyto-domain to activate downstream signaling pathways. To understand the signal transduction mechanism of syndecan, it is important to know the interactions between their actual structure and function in vivo. Therefore, it is important to identify the structure of SDC4 to understand the ligand binding behavior of SDC4. In this study, expression and purification were performed to reveal structures of the short ecto-domain, the transmembrane domain, and the cytoplasmic domain of Syd4-eTC (SDC4). Solution-state NMR spectroscopy and solid-state NMR spectroscopy were used to study the structure of Syd4-eTC in membrane environments and to demonstrate the interaction between Syd4-eTC and PIP_2_.

## 1. Introduction

Syndecans are a major family of cell surface heparan sulfate proteoglycans (HSPGs) consisting of core proteins and heparan sulfate and chondroitin sulfate [1,2,3]. There are a family of four (Syndecan-1, -2, -3 and -4) and one of them, syndecan-4, is expressed in most cell types of mammals, unlike the rest of family, which has limited tissue distribution [4,5,6]. The syndecan protein family has an extracellular domain with an ecto-domain, a transmembrane domain, and a cytoplasmic domain in common [2,7]. The ectodomain is composed of heparan sulfate and chondroitin sulfate chains that can interact with various ligands. This is the most unique region among syndecan family proteins and has a diverse length and sequence homology [8]. Their cyto-domain, excluding the transmembrane domain, consists of hydrophobic amino acids divided into C (conserved) 1, V (variable), and C2 domains [9]. The V region is a very heterogeneous part of the four mammalian syndecans, and due to these characteristics, it is the most extensively studied part of syndecan-4 [5]. On the other hand, the C1 and C2 regions do not show significant differences in amino acid sequence in the mammalian syndecan family [5].

Syndecan-4 (SDC4) performs various functions, including cell-to-cell interactions, extracellular matrix interactions, growth factor–receptor activation, and matrix adhesion [2,10,11,12]. As such, SDC4 participates in several signaling pathways and functions as a structural protein. Interestingly, SDC4 is overexpressed when tumor cells form. It has been reported that the overexpression of SDC4 in tumorigenesis is stimulated by tumor-suppressor molecules. Such overexpression can revert the cell-adhesion defect of tenascin-C, which enhances tumor proliferation [13,14].

Regarding the overall cellular function of SDC4, other cells or ligands can bind to its ecto-domain via heparan sulfate glycosaminoglycan (HS-GAG). In addition, there is also a ligand attached to the V domain of SDC4, 4,5-bisphosphate phosphatidylinositol (PIP_2_). PIP_2_, a membrane phospholipid found in all eukaryotic cells, interacts with SDC4 to regulate oligomerization of the SDC4 cytoplasmic domain and also regulates SDC4-mediated protein kinase C (PKC) activation [15,16]. The SDC4 dimer binds to the catalytic subunit of PKCα by binding to two PIP_2_ molecules [8,17,18,19,20,21], and the resulting activation complex is regulated by phosphorylation of cytoplasmic Ser179 of SDC4 [22]. This alters the conformation of the C2 region of the cytoplasmic domain, causing loss of PIP_2_ binding and ultimately leading to a lack of PKCα activation [17,23]. In addition, the oligomerization of SDC4 is known to govern the function of SDC4 [24,25,26], and communication between the cyto-domain and ecto-domain is known to be mediated by structural changes in the transmembrane domain [27].

It is known that PIP_2_ exerts a regulatory effect on the function of SDC4, but it is not yet well-known which amino acid sequence of SDC4 interacts with PIP_2_. Therefore, there are few reports on the overall structure of SDC4 except for its cyto-domain [23,24,28]. To understand the signaling mechanism of SDC4, it is important to know the relationship between the actual structure of the domain and its in vivo function. Based on this knowledge, new therapeutic strategies can be developed to regulate signal transduction and treat tumor cell metastasis [27].

Among the many spectroscopic methods, NMR spectroscopy is a suitable tool for investigating the structure of transmembrane proteins in the membrane environment. In our previous study, we were able to reveal that the secondary structure of the transmembrane domain of SDC4, obtained through optimized expression and purification procedures, was an alpha helix through CD and solution NMR spectroscopy [29]. Also, due to the GXXXG motif in the transmembrane domain of SDC4, it was confirmed through solution/solid-state NMR that dimerization can be formed [30,31,32,33]. Additionally, the GXXXG motif is present in the transmembrane region of syndecan-2, which is highly similar to the transmembrane domain of SDC4, as well as in syndecan-1 and -3 [3]. In particular, it is known that the transmembrane domain of syndecan-2 forms an alpha helix and dimerization in detergent micelles [34].

In this study, to study the interaction between SDC4 and PIP_2_, NMR spectroscopy was applied in the presence of DPC micelles, and we used Syd4-eTC (consisting of a small ecto-domain, a full transmembrane domain, and a full cytodomain) expressed in a previous study [30]. We attempted to use chemical shift perturbation (CSP) to confirm which residues of SDC4 interact with PIP_2_. In solid-state NMR, bicelle can be used to investigate the intrinsic structure of a transmembrane protein. The bicelle aligned with a magnetic field is a suitable medium to study the structure of a transmembrane protein with solid-state NMR spectroscopy. A solid-state NMR probe for ^1^H-^15^N measurement was designed and manufactured in our laboratory to identify the unique tertiary structure and topology of Syd4-eTC in an environment mimicking the cell membrane. The 2D solid-state NMR spectroscopy with a separate local field type experiment was used to analyze the intrinsic structure of Syd4-eTC on bicelle, and the Polarity Index at Slanted Angle (PISA) wheel pattern was used to analyze the tilt angle of transmembrane helix [35,36,37].

## 2. Results and Discussion

### 2.1. Solution-State NMR Spectroscopy

**Figure 1:**
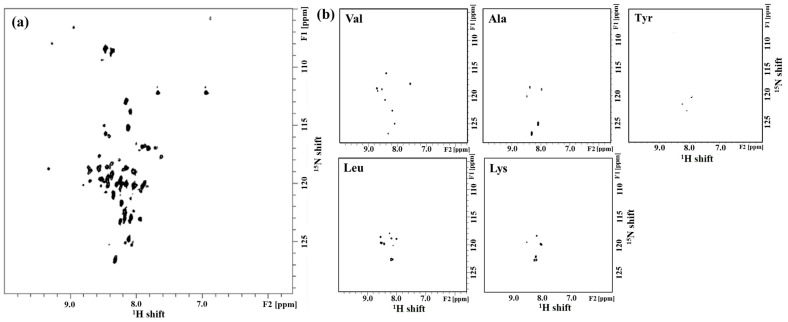
(**a**) ^1^H-^15^N 2D HSQC spectra of uniformly ^15^N-labeled Syd4-eTC and (**b**) ^1^H-^15^N 2D HSQC spectra of selectively ^15^N-labeled Syd4-eTC (Val, Ala, Tyr, Leu, Lys). The ^1^H-^15^N 2D HSQC spectra of Syd4-eTC were measured with 100 mM DPC micelle in 90% H_2_O/10% D_2_O at 323 K and pH 4.0.

Figure 1a shows ^1^H-^15^N 2D HSQC spectra of Syd4-eTC. All cross-peaks were well dispersed, and two or more resonance pairs of asparagine side chains were observed at 112 ppm. This means that the structure of Syd4-eTC is predominantly α-helix and can have a multimeric form in DPC micelles [30]. The cross-peaks in the ^1^H-^15^N HSQC spectrum of a ^15^N-labeled peptide represent all backbone amide groups of the peptide. Therefore, if the number of cross-peaks in the ^1^H-^15^N HSQC spectrum matches the number of amino acid sequences in the peptide, this can indicate that a highly pure protein has been obtained, and can be considered to take the form of a monomer on DPC micelles. 

The number of amino acids of Syd4-eTC is 57, but as a result of the HSQC spectrum, the number of peaks exceeded about 60. One of the reasons for this is as follows. In the case of ASN, there is only one in the amino acid sequence of Syd4-eTC, and the appearance of one additional ASN peak in the region in HSQC is judged to mean that a dimer was formed. It is believed that these results are reflected by the large number of peaks in HSQC. The ^1^H-^15^N 2D HSQC spectra of Syd4-eTC for selectively ^15^N-labeled (Val, Ala, Try, Leu, Lys) Syd4-eTC are shown in Figure 1b. Cross-peaks of uniformly ^15^N-labeled HSQC spectrum can be assigned based on the ^1^H-^15^N 2D HSQC spectra of selectively labeled peptides and 2D HMQC-NOESY spectra of uniformly labeled peptides. 

**Figure 2:**
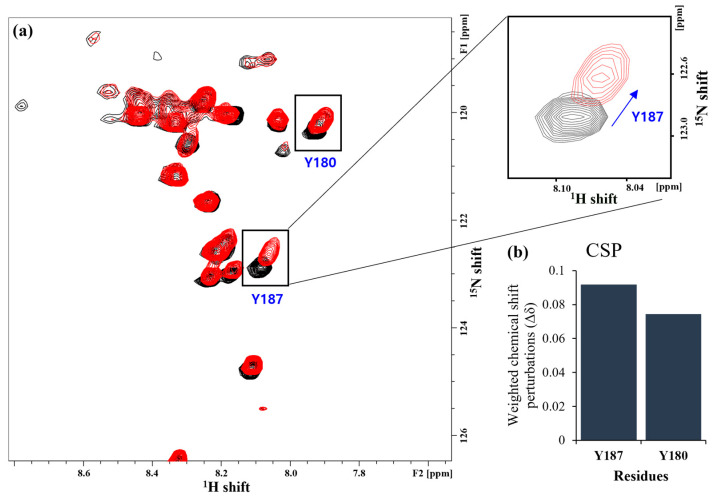
(**a**) ^1^H-^15^N 2D HSQC spectra for Syd4-eTC with/without PIP_2_ (molar fractions of 1:1). Uniformly ^15^N-labeled ^1^H-^15^N 2D HSQC spectrum for Syd4-eTC (black) and uniformly ^15^N-labeled ^1^H-^15^N 2D HSQC spectra for Syd4-eTC mixed with PIP_2_ by molar fractions of 1:1 (red). (**b**) Chemical shift perturbation (CSP) plots for each are also shown below. When compared to the uniformly ^15^N-labeled ^1^H-^15^N 2D HSQC spectra of Syd4-eTC, one cross-peak noticeably shifted. In addition, when compared with the selectively ^15^N-labeled ^1^H-^15^N 2D HSQC spectrum, it was confirmed that the shifted cross-peak was Tyrosine. The ^1^H-^15^N 2D HMQC-NOESY experiment of uniformly ^15^N-labeled peptide was used to confirm that this was the 187th tyrosine of the cyto-domain. The chemical shift perturbation that occurred in Y187 is indicated by a blue arrow in the inset.

Figure 2a shows the ^1^H-^15^N 2D HSQC spectra for Syd4-eTC with/without PIP_2_ (molar fractions of 1:1). Through this spectrum, the uniformly ^15^N-labeled ^1^H-^15^N 2D HSQC spectrum, the selectively ^15^N-labeled ^1^H-^15^N 2D HSQC spectrum, and the ^1^H-^15^N 2D HMQC-NOESY spectrum, the residue where the chemical shift perturbation (CSP) of the cross-peak occurs was confirmed. For this, CSP analysis, NMRFAM-SPARKY, CCPN analysis, and NMR box programs were used, and CSP was calculated using the following equation [38,39,40]:

(1) $${CSP}_{i}=\sqrt{{\left({∆\delta }_{Hi}\right)}^{2}+\alpha {\left({∆\delta }_{Ni}\right)}^{2}}$$

Here, there overall chemical shift patterns of peaks in the uniformly ^15^N-labeled ^1^H-^15^N 2D HSQC spectrum and the ^1^H-^15^N 2D HSQC spectrum where Syd4-eTC and PIP_2_ coexist seem to be almost similar. However, when observing the enlarged spectrum, there was a portion in which CSP occurred, and the cross-peak where CSP occurred was confirmed to be a tyrosine residue (Tyr187) in the V domain of Syd4-eTC. In addition, it was confirmed that some CSP occurred in Tyr180 outside the V domain. Another thing that could be found in the spectrum was that, even though PIP_2_ was bound to the cyto-domain of Syd4-eTC, the cross-peak CSP corresponding to the transmembrane domain residues could not be confirmed in the ^1^H-^15^N 2D HSQC spectrum.

### 2.2. Solid-State NMR Spectroscopy

**Figure 3:**
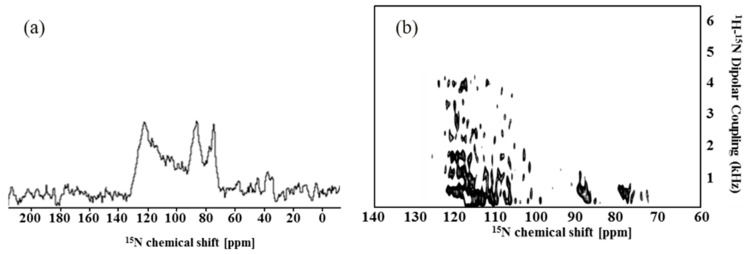
1D ^1^H-^15^N CP solid-state NMR spectrum (**a**) and 2D ^1^H-^15^N SAMMY spectrum (**b**) of uniformly ^15^N-labeled Syd4-eTC. Syd4-eTC is magnetically aligned in the bicelle. The relative position of the protein can be determined by the resonance position and the region of the spectrum. In addition, the 2D SAMMY spectrum with a characteristic PISA wheel-like pattern provides information on the slanted angle via the calculated PISA wheel pattern.

^15^N resonance of solid-state NMR 1D ^1^H-^15^N cross-polarization (CP) spectrum can show an interaction between a peptide and a membrane. The bicelle consisting of long-chain and short-chain phospholipids is easily oriented to one side along the magnetic field, which is an unflipped bicelle. In the solid-state NMR 1D ^1^H-^15^N CP spectrum of Syd4-eTC on the unflipped bicelle, a peak appeared over the range of 70–130 ppm (Figure 3a). In the case of the unflipped bicelle, peptides placed on the surface domain of bicelle showed peaks in the field that were lower than 120 ppm, while peptides placed in the transmembrane domain of the bicelle showed peaks in the field higher than 120 ppm [41]. This meant that Syd4-eTC in magnetically aligned bicelles underwent both rotational diffusion about the parallel axis to the magnetic field due to the transmembrane domain of peptide and rotational diffusion about the perpendicular axis to the magnetic field due to the cyto-domain of the peptide.

The sandwich-based separated local field spectroscopy (2D SAMMY) spectrum provides more specific information than the 1D spectrum. The structure of Syd4-eTC in a membrane environment was determined by two orientation-dependent frequencies, ^15^N chemical shift and ^1^H-^15^N dipolar coupling constant. The 2D SAMMY spectrum provides an indication of the topology of the peptide in the membrane. Moreover, the SAMMY spectra of uniformly ^15^N-labeled peptides have a characteristic PISA wheel pattern that provides a direct measurement of helix tilt and rotation [37,42]. The 2D SAMMY spectrum of Syd4-eTC is shown in Figure 3b. Many cross-peaks of the ^1^H-^15^N dipolar coupling were observed within the range of 2–4.5 kHz. This dipolar coupling is evidence of transmembrane helix [37,43]. Also, cross-peaks in the dipolar coupling range below 2 kHz are presented by proteins on the surface of the biological membrane.

**Figure 4:**
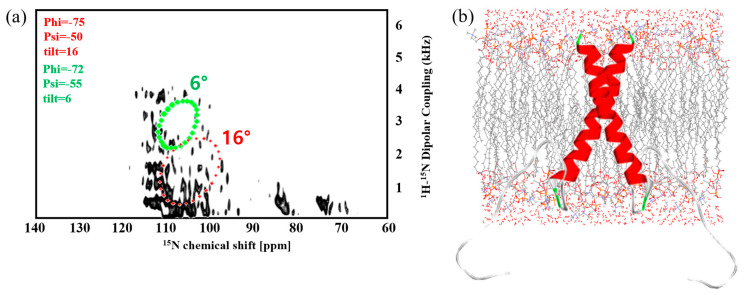
2D ^1^H-^15^N SAMMY spectrum that overlaps PISA wheel simulation of uniformly ^15^N−labeled Syd4-eTC (**a**) and structure of Syd4-eTC in membrane environments (**b**). PISA wheel pattern showed peptide tilt angle according to the normal bilayer. The color representation is based on the secondary structure: Red is helix, green is turn, and white is random coil. ^1^H-^15^N solid-state NMR experiments were performed using a home-built ^15^N-^1^H SSNMR probe with a 5 mm strip of shielded solenoidal rf coil for a 400 MHz narrow bore magnet.

PISA wheel patterns were analyzed based on the 2D SAMMY spectrum of Syd4-eTC. The PISA wheel pattern overlapped with the 2D SAMMY spectrum. To estimate the structure of the peptide in the 2D SAMMY spectrum, it was compared with numerical simulation. The PISA wheel pattern superimposed on 2D SAMMY data of Syd4-eTC is shown in Figure 4a. Based on the presence of the PISA wheel bound, tilt angles of Syd4-eTC were determined to be 6° and 16°. The three-dimensional structure of Syd4-eTC has a slightly curved α-helix structure. This was generated using homology modeling with template proteins. The thickness of the membrane is 23 Å. This corresponds to the thickness of the bicelle. The dimeric structure is formed by the GXXXG motif in the transmembrane domain. The two monomers have different orientations for the membrane [24]. A suitable conformer of Syd4-eTC obtained through MD simulation has tilt angles of 6° and 16°, consistent with the PISA wheel pattern analysis based on the ^1^H-^15^N 2D SAMMY solid-state NMR spectrum. The lipid bilayer complex visualized by CHARMM-GUI and the best conformer obtained from MD simulations are shown in Figure 4b.

## 3. Materials and Methods

### 3.1. Expression and Purification of Syd4-eTC

The oligonucleotide encoding the Syd4-eTC was synthesized by Integrated DNA Technologies (Coralville, IA, USA) and cloned into the pET31b(+) expression vector (Novagen, Madison, WI, USA). The amino acid sequence of Syd4-eTC used here is ERTEV LAALI VGGVV GILFA VFLIL LLVYR IKKKD EGSYD LGKKP IYKKA PTNEF YA, which contains 57 residues. To express a large amount of protein, a pre-culture step was performed using LB medium with carbenicillin. A fully grown culture was added to M9 minimal medium at a volume of 1%, and culture was grown overnight at 37 °C, with shaking at 230 rpm. At this time, ^15^N-enriched ammonium sulfate (Cambridge Isotope Lab, Andover, MA, USA) was used to prepare uniformly ^15^N-labeled peptides for structural analysis of Syd4-eTC. When preparing selectively ^15^N-labeled peptides, ^15^N-labeled amino acid (Cambridge Isotope Lab, USA) along with other unlabeled 19 amino acids were used to make M9 minimal medium. A total of 1 M IPTG was added when OD_600_ value was at 0.5 to induce overexpression of the Syd4-eTC, and cells were incubated at 37 °C with shaking at 230 rpm for about 16 h. The expressed cells were harvested into pellet by centrifugation (6000× *g*, 4 °C, 30 min) and stored at −80 °C. 

The stored cell pellet was resuspended and lysed using lysis buffer (20 mM Tris, 500 mM NaCl, 15% glycerol) with lysozyme (0.5 mg/mL; Sigma-Aldrich, St. Louis, MO, USA). Cells were mechanically disrupted through ultrasonication, and cell lysate was centrifuged at 4 °C 14,500 rpm for 30 min to obtain fusion proteins. Ketosteroid isomerase (KSI)-fusion proteins were obtained by pellet and these insoluble aggregates were dissolved in Ni-NTA binding buffer (20 mM Tris, 500 mM NaCl, 5 mM Imidazole, 6 M Guanidine-HCl, pH 7.9–8.0) for 5 h or more. Afterwards, the supernatant was centrifuged (4 °C, 14,500 rpm, 30 min) to remove remaining impurities and applied to Ni-NTA affinity chromatography. To prepare Ni-NTA column, charging buffer (50 mM NiSO_4_·6H_2_O) was flowed to bind Ni^2+^ to NTA agarose resin, and then washed with ddH_2_O to remove unbound Ni^2+^. Binding buffer was flowed to calibrate the resin and apply the sample to the column prepared in this way. Remaining cell components were removed with a washing buffer (20 mM Tris, 500 mM NaCl, 16 mM Imidazole, 6 M Guanidine-HCl, pH 7.9–8.0). The KSI-fusion proteins with His_6_-tag were then eluted with an elution buffer (20 mM Tris, 500 mM NaCl, 500 mM Imidazole, 6 M Guanidine-HCl, pH 7.9–8.0) containing excess imidazole. Elutes were dialyzed with ddH_2_O for 1 day using a dialysis bag with molecular weight cut-off (MWCO) of 10 kDa (Spectrum Labs, Rancho Dominguez, CA, USA) to remove denaturant and salts. After completing the dialysis step, the fusion protein was precipitated in the dialysis bag and collected in fluffy form after lyophilization. To separate the fusion partner, KSI and His_6_-tag, chemical cleavage was performed for 5 h in a dark room using 70% formic acid (Sigma-Aldrich, USA) and CNBr (Sigma-Aldrich, USA) to cut off the methionine residue. After cleavage, dialysis was performed using a dialysis bag with MWCO of 1 kDa and ddH_2_O to remove CNBr and formic acid. Lyophilization was carried out to obtain the protein in fluffy powder form. In each purification step described above, 12% Tris-Tricine SDS PAGE was conducted to check whether protein expression, isolation, and purification proceeded properly. Lyophilized protein mixture was purified using a semi-preparative reverse-phase HPLC C18 column (Phenomenex, Jupiter, Torrance, CA, USA) and a Delta 600 HPLC system (Waters, Milford, MA, USA). Eluent A consisted of 95% H_2_O, 5% ACN, and 0.1% TFA. Eluent B consisted of 95% ACN, 5% H_2_O, and 0.1% TFA. Using a gradient elution method, the composition of eluent B was increased by 2% per minute until 5–35 min, and 1% per minute until 35–80 min. The flow rate was set at 3 mL/min for 90 min. The protein mixture was dissolved in eluent A at 2 mg/mL. After sonication to dissolve the protein mixture, the solution was centrifuged at 14,500 rpm for 30 min at 4 °C to remove impurities. Using a photodiode array (PDA), peak fractions were detected at wavelengths of 220 nm and 280 nm. Each peak fraction was confirmed by 12% Tris-Tricine SDS PAGE, and the final purified peptide was obtained by lyophilization.

### 3.2. Solution-State NMR Spectroscopy

A Bruker Avance III HD 400 MHz narrow bore NMR spectrometer at 9.4 Tesla (Bruker Biospin, Rheinstetten, Germany) was used to perform NMR measurements on the DPC micelle of Syd4-eTC. A total of 0.5 mg of Syd4-eTC labeled with ^15^N was dissolved in D_2_O/H_2_O 1:9 solvent, and 100 mM DPC (Cambridge Isotope Laboratories, Andover, MA, USA) and 10X NMR salt (100 mM Na_2_HPO_4_, 10 mM NaN_3;_ 0.1% of the total volume) were added. Samples of Syd4-eTC and PIP_2_ were prepared at molar fractions of 1:1 on 100 mM DPC micelle, respectively, to analyze the structural characteristics of the interaction between Syd4-eTC and PIP_2_. The experiment was conducted at 323 K and pH 4.0. 2D ^1^H-^15^N heteronuclear single quantum coherence spectroscopy (HSQC) experiment with uniformly/selectively ^15^N-labeled Syd4-eTC was performed with complex points of the t1/t2 increments set to 256 and 2048, respectively, with 64 transients. To assign resonances of the HSQC spectrum, a 2D heteronuclear multiple quantum correlation-nuclear overhauser effect spectroscopy (HMQC-NOESY) experiment was performed with complex points of the t1/t2 increments set to 256 and 1024, respectively, with 32 transients. The mixing time was set to 300 ms. All spectra were processed with Bruker topspin 4.0.7 software (Bruker Biospin, Rheinstetten, Germany).

### 3.3. Solid-State NMR Spectroscopy

Experiments were performed using a Bruker Avance III HD 400 MHz NB NMR spectrometer at 9.4 Tesla (Bruker Biospin, Rheinstetten, Germany) with a Z-gradient unit. Solid-state NMR was used to study the three-dimensional structure of transmembrane protein in membrane system. All experiments for NMR measurements from magnetically aligned bicelles with Syd4-eTC were carried out using an ^1^H-^15^N double-resonance, home-built, solid-state NMR probe with a solenoidal coil 400 MHz standard bore magnet.

A 400 MHz narrow-bore ^1^H-^15^N double-resonance home-built solid-state NMR probe was designed and constructed for oriented biological samples. All probe compositions were made of non-magnetic materials. The probe body comprised a 6061 aluminum pipe and was refined to an outer diameter (O.D) of 39.5 mm and an inner diameter (I.D) of 39.1 mm. Probes with a 5 mm strip shielded solenoidal rf coil were made using Cross–Waugh type circuits for a double-tuned configuration due to its good isolation of frequencies. The tuning ranges of the high-side channel and the low-side channel were 395–404 MHz, 39.1–42 MHz, respectively. The 1D and 2D NMR spectrum of standard reference NAL (^15^N-acetyl-leucine) was obtained for Bruker Avance III HD 400 MHz NB NMR spectrometer. 

Bicelle was q = 3.2 using long-chain 14-O-PC and short-chain 6-O-PC using 3 mg of Syd4-eTC. This protein was analyzed to determine the PISA wheel pattern, tilt angle, and average dihedral angle of the transmembrane protein using 1D ^1^H-^15^N CP and SAMMY technique. Results were compared with those obtained with the computational calculation method.

In 1D ^1^H-^15^N cross-polarization (CP) experiments, 90° pulse length was set at 5.5 μs and CP contact time was set at 5 ms. ^15^N chemical shift reference was based on ammonium sulfate (AMS; 26.8 ppm). The complex point was set to be 1024. The number of scans was 8192. The line-broadening was set to be 20 Hz and the zero-filling point was set to be 2048.

The 2D magic-sandwich-based separated local-field spectroscopy (SAMMY) solid-state NMR experiment was conducted at room temperature. The 90° pulse length was set to be 5.5 μs. CP contact time was 5 ms. The t_1_ increment was 28 and t_2_ scan was 1024. The t_2_ complex point was set to 512. The ^1^H offset frequency for heteronuclear decoupling was about 9.5 ppm. The line broadening was adjusted to 20 Hz. The zero-filling point was adjusted to 2048 for the F2 dimension and 256 for the F1 dimension. All 1D solid NMR data and 2D solid NMR data were obtained using Bruker Topspin 3.5 software and NMRPipe/NMRDraw.

PISA wheel pattern was calculated using MATLAB and SIMULINK R2010a (MathWorks, Natick, MA, USA) [44,45]. The ^1^H-^15^N 2D SAMMY spectrum of Syd4-eTC in bicelle showed a characteristic circular pattern. This pattern, also called the PISA index, was named because the resonance shape looks similar to the projection boundary and shadows of the Leaning Tower of Pisa. In this calculation, principal values of σ_11_ = 64 ppm, σ_22_ = 77 or 88 ppm for ^15^N, and σ_11_ = 3 ppm, σ_22_ = 8 ppm, σ_33_ = 17 ppm for ^1^H were used for chemical shift tensors, respectively. This numerical simulation used the order parameter of bicelle S = 0.80–0.85. The optimized torsion angle was Φ = −69° and Ψ = −42°.

In a previous report, the structure of the transmembrane domain of SDC4 was revealed using molecular dynamics (MD) simulation [24]. Syd4-eTC can also form a dimeric structure because SDC4 can form a dimer via the GXXXG motif. In the present study, homology modeling with multiple sequence alignments was introduced and homologous proteins of Syd4-eTC were retrieved by PSI-BLAST. Multiple sequence alignment was performed to align Syd4-eTC with template proteins using 1EJQ, 2M7G, 3RLB, 2B2H, and 4J7C. The actual model of the membrane was generated by MD simulation. At this time, an NVT (constant-temperature and constant-volume) statistical ensemble model was used for the thermodynamic ensemble. The dimeric structure of Syd4-eTC was simulated using the “Dock proteins (ZDOCK)” protocol. This is a basic component of the protein–protein docking scoring function. It uses a geometric descriptor based on surface curvature of surface area. After the dimerization of Syd4-eTC, the CHARMM36 all-atom empirical force field (c39b1) was used for all calculations. A generalized-born with simple switching (GBSW) solvation model was used to optimize the position and orientation of Syd4-eTC in the implicit membrane using the ‘Add membrane and molecules’ protocol. The membrane thickness was calculated to be 23 Å. Improvements were made using the energy-minimization method with the SHAKE constraint algorithm as a constraint routine to remove the fastest degrees of freedom for hydrogen-containing bonds. All modeling and calculations in this study were performed using Discovery studio 2016 (Biovia, San Diego, CA, USA). Lipid bilayer complexes were drawn using membrane builder modules from CHARMM-GUI (http://www.charmm-gui.org/, accessed on 13 November 2018). The results of the CHARMM-GUI membrane builder module were visualized with Discovery studio.

## 4. Conclusions

The structure of Syd4-eTC in the membrane was analyzed using solution-state NMR and solid-state NMR. Based on the plot of the chemical shift perturbation of spectra of uniformly labeled ^1^H-^15^N 2D HSQC, selectively labeled ^1^H-^15^N 2D HSQC, and uniformly labeled ^1^H-^15^N 2D HMQC-NOESY demonstrated that PIP_2_ could bind to the 187th tyrosine of the cyto-domain of SDC4. Solid-state NMR experiments were performed to clarify the three-dimensional structure of Syd4-eTC in membrane environments. The PISA wheel pattern was analyzed based on ^1^H-^15^N 2D SAMMY solid-state NMR spectrum. The structure of Syd4-eTC in membrane environments was confirmed by using molecular dynamics (MD) simulation and the CHARMM-GUI membrane builder module was visualized with Discovery studio.

## Data Availability

Data are contained within the article.
